# Crystal structure of (1′*S*,2′*S*,3*S*)-1′-benzoyl-2′-(4-meth­oxy­phen­yl)-1-methyl-2′,5′,6′,10b′-tetra­hydro-1′*H*-spiro­[indoline-3,3′-pyrrolo­[2,1-*a*]isoquinolin]-2-one

**DOI:** 10.1107/S2056989020010300

**Published:** 2020-09-04

**Authors:** Janet Priyavathani Selvaraj, Stella Mary, Jyoti Boruah Dhruba, Birkumar Singh Huidrom, Yuvaraj Panneerselvam, Kannan Piskala Subburaman

**Affiliations:** a Department of Physics, St.Peter’s University, Avadi, Chennai-600054, Tamilnadu, India; b Applied Organic Chemistry Group, Chemical Science and Technology Division, CSIR-North East Institute of Science and Technology, Jorhat-785006, India; c Academy of Scientific and Innovative Research (AcSIR), CSIR-NEIST Campus, India; d CSIR-North East Institute of Science and Technology (NEIST), Branch Laboratory, Lamphepat-795004, Imphal, Manipur, India; e Department of Physics, Kings Engineering College, Irungattukottai, Sriperumbudur, Chennai–602117, Tamilnadu, India

**Keywords:** crystal structure, spiro, indoline, tetra­hydro­iso­quinoline, pyrrolidine

## Abstract

In the title spiro compound, C_34_H_30_N_2_O_3_, the central pyrrolidine ring is fused with the tetra­hydro­iso­quinoline ring, both having distorted envelope conformations, with the flap atoms being C and N, respectively. The meth­oxy­phenyl group is attached to the pyrrolidine ring, and is disordered over two positions, with refined occupancies of 0.638 (6):0.362 (6) Å. The central pyrrolidine ring is inclined relative to the tetra­hydro­iso­quinoline group, such that the dihedral between the non-flap atoms of each ring system is 11.29 (7)°. The spiro-linkage creates a dihedral angle of 83.26 (5)° between the indolinone ring and the non-flap atoms of the pyrrolidine ring. In the crystal, mol­ecules are linked *via* C—H⋯O hydrogen bonds. For the major disorder component, these form *C*(11) chains that propagate parallel to the *a* axis.

## Abstract 2

The synthesis and crystal structure of the spiro title compound is presented.

## Chemical context   

Spiro frameworks are often utilized in drug design as a result of their three-dimensionality and structural diversity, which provide a framework for the attachment of pharmaceutically relevant active sites (Kobayashi *et al.*, 1991 ▸). The spiro-pyrrolidine structural motif is present in numerous naturally occurring and pharmacologically important alkaloids. The spiro-pyrrolidine-indolin-2-one framework in particular is found in a number of alkaloids of biological significance (Hilton *et al.*, 2000 ▸). Some of these compounds have been used as anti­microbial and anti­tumour agents (Sundar *et al.*, 2011 ▸), or have analgesic (Crooks & Sommerville, 1982 ▸) and anti-influenza properties (Stylianakis *et al.*, 2003 ▸). Taking into account the significance of spiro compounds in this context, the single-crystal X-ray structure of the title compound, **1**, was determined.
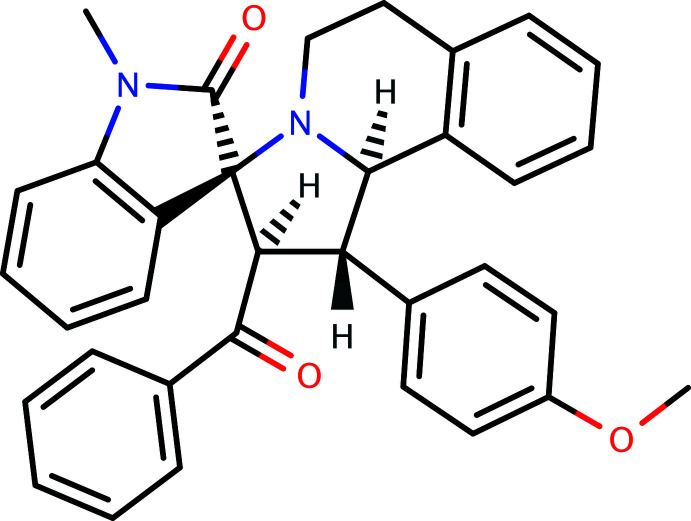


## Structural commentary   

**Figure 1:**
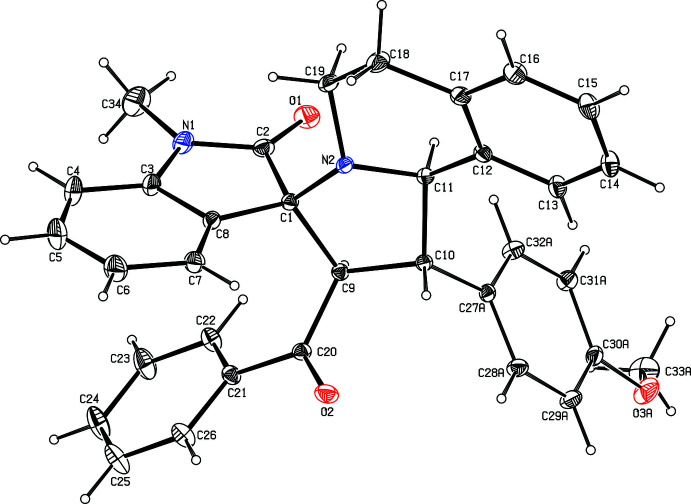
A view of the title compound showing the atom-labelling scheme. Displacement ellipsoids are drawn at the 30% probability level. For the sake of clarity, the minor component of disorder of the meth­oxy­phenyl group is not shown.

An ellipsoid plot of **1** is shown in Fig. 1 ▸. The bond lengths (Allen *et al.*, 1998 ▸) and bond angles are all normal. The tetra­hydro­iso­quinoline (N2/C11–C19) and pyrrolidine (N2/C1/C9–C11) rings each have envelope conformations, with the maximum deviation of the flap atoms being −0.363 (1) Å (for N2) and 0.253 (2) Å (for C11), respectively. These non-planar ring systems are fused at N2/C11, such that the dihedral angle between their non-flap atoms is 11.29 (7)°. The *N*-methyl indolinone ring system (N1/C1–C8/O1/C34) is essentially planar, with the maximum deviation being 0.030 (2) Å for the oxygen atom (O1). This group is attached to the pyrrolidine ring *via* the spiro-linkage, forming a dihedral angle with the non-flap atoms (N2/C1/C9/C10) of the pyrrolidine of 83.26 (5)°. The sum of the bond angles around N1 and N2 (360.0 and 338.33°, respectively) is in accordance with *sp* ^2^ and *sp* ^3^ hybridization states (Beddoes *et al.*, 1986 ▸).

The meth­oxy­phenyl group is disordered over two orientations, with refined occupancies of 0.638 (6):0.362 (6). The disordered meth­oxy­phenyl ring major (C27–C33/O3) and minor (C27*D*–C33*D*/O3*D*) components are largely planar, with the maximum deviations from their respective mean planes being observed for the methyl carbons, C33 [0.303 (7) Å] and C33*D* [0.130 (12) Å].

## Supra­molecular features   

**Figure 2:**
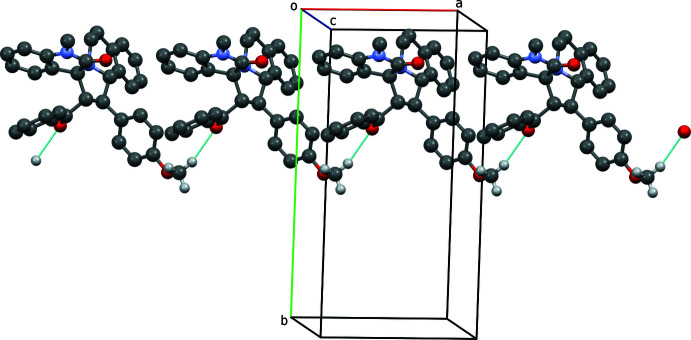
The crystal packing of the title compound, showing the *C*(11) chain (major disorder component only) running parallel to the *a* axis. Hydrogen bonds are shown as dotted lines. Only the H atoms on groups involved in the hydrogen bonding are included.

In the crystal packing of **1**, there are no classical hydrogen bonds or π–π inter­actions between the various rings of adjacent mol­ecules. There are, however, different weak C—H⋯O close contacts for the two disorder components (Table 1 ▸). For the major component, there is a close contact between translation-related mol­ecules, C33—H33*B*⋯O2^i^, of 3.490 (5) Å [symmetry code: (i) *x* + 1, *y*, *z*], while for the minor component there is a close contact between glide-related mol­ecules, C4—H4⋯O3^ii^, of 3.559 (6) Å [symmetry code: (ii) *x* − 1, −*y* + 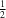, *z* − 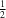]. For the major component, these generate *C*(11) chains (Bernstein *et al.*, 1995 ▸) that propagate parallel to the *a* axis (Fig. 2 ▸).

## Database survey   

A search in the Cambridge Structural Database (CSD, version 5.39, update August 2018; Groom *et al.*, 2016 ▸) using pyrrolidine as the search fragment produced over 11600 hits. For the core spiro-pyrrolidine/*N*-methyl pyrrolidone fragment, the yield was a more modest 88 hits. These 88 structures show many different substitution patterns. The four structures with the most features in common with **1** are probably RAQCIY (Du *et al.*, 2017 ▸), IFETAR (Guo *et al.*, 2018 ▸), DOHMEV (Boudriga *et al.*, 2019 ▸), and KIFRID (Zhang *et al.*, 2018 ▸), though none of these are especially similar to **1**.

## Synthesis and crystallization   

In a 50 mL round-bottom flask, 1-methyl­isatin (0.5 mmol) was dissolved in toluene (5 mL) followed by the addition of 1,2,3,4-tetra­hydro­iso­quinoline (0.5 mmol) and the mixture was stirred at room temperature for half an hour. After that, (*E*)-3-(4-meth­oxy­phen­yl)-1-phenyl­prop-2-en-1-one (0.5 mmol) was added to the reaction mixture and stirring was continued at 383 K for 10 h. The reaction was monitored for the formation of the product by TLC at regular inter­vals. Soon after the formation of the product, the reaction mixture was concentrated under reduced pressure and extracted with ethyl acetate/water (*v*/*v* = 75:25). The organic layer was dried over anhydrous sodium sulfate and concentrated under vacuum to yield the crude product, which was purified by column chromatography using ethyl acetate/*n*-hexane (3:17) as eluent. 0.2g of the compound were dissolved in ethanol and the solution was kept undisturbed in the open air for one week. After five days, crystals started to appear and were separated carefully.

**^1^H NMR** (500 MHz, CDCl_3_) δ 7.60 (*d*, *J* = 8.7 Hz, 2H), 7.28–7.22 (*m*, 3H), 7.14–7.03 (*m*, 5H), 7.02–6.98 (*m*, 1H), 6.96–6.90 (*m*, 3H), 6.92–6.85 (*m*, 1H), 6.75 (*d*, *J* = 7.8 Hz, 1H), 6.32 (*d*, *J* = 7.7 Hz, 1H), 5.15 (*d*, *J* = 10.1 Hz, 1H), 4.52 (*d*, *J* = 9.7 Hz, 1H), 4.24 (*t*, *J* = 9.9 Hz, 1H), 3.81 (*s*, 3H), 3.07 (*s*, 3H), 2.92 (*d*, *J* = 7.7 Hz, 2H), 2.65 (*d*, *J* = 12.3 Hz, 1H), 2.52–2.47 (*m*, 1H).

**^13^C NMR** (126 MHz, CDCl_3_) δ 197.0, 178.63, 158.55, 143.58, 138.24, 137.45, 134.71, 133.83, 132.39, 130.02, 129.07, 128.78, 127.77, 127.46, 127.12, 126.26, 126.15, 125.46, 125.04, 123.04, 114.43, 107.16, 70.87, 64.13, 63.59, 55.26, 49.83, 42.39, 30.32, 25.89.

## Refinement   

Crystal data, data collection and structure refinement details are summarized in Table 2 ▸. Hydrogen atoms were positioned geometrically (C—H = 0.93–0.98 Å) and allowed to ride on their parent atoms, with *U*_iso_(H) = 1.5*U*_eq_(C) for methyl H and 1.2*U*_eq_ (C) for other H atoms. The occupancies of the disorder group of the meth­oxy phenyl moiety were initially allowed to ride then it was fixed and refined. The benzene rings were refined as rigid hexa­gons with C—C distances of 1.39 Å. The other bond lengths of the major and the minor components were made similar using similarity restraints with an s.u. of 0.01 Å. The positions of the meth­oxy­phenyl moiety (C30/O3/C33) atoms are disordered over two positions with site occupancy factors of 0.638 (6) and 0.362 (6), respectively.

## Supplementary Material

Crystal structure: contains datablock(s) I. DOI: 10.1107/S2056989020010300/tx2027sup1.cif

Structure factors: contains datablock(s) I. DOI: 10.1107/S2056989020010300/tx2027Isup2.hkl

Supporting information file. DOI: 10.1107/S2056989020010300/tx2027Isup3.cml

CCDC reference: 1983982

Additional supporting information:  crystallographic information; 3D view; checkCIF report

## Figures and Tables

**Table 1:** Hydrogen-bond geometry (Å, °)

*D*—H⋯*A*	*D*—H	H⋯*A*	*D*⋯*A*	*D*—H⋯*A*
C33—H33*B*⋯O2^i^	0.96	2.61	3.490 (5)	153
C4—H4⋯O3*D* ^ii^	0.93	2.65	3.559 (6)	166

Symmetry codes: (i) 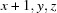; (ii) 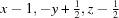.

**Table 2:** Experimental details

Crystal data	
Chemical formula	C_34_H_30_N_2_O_3_
*M*_r_	514.60
Crystal system, space group	Monoclinic, *P*2_1_/*c*
Temperature (K)	294
*a*, *b*, *c* (Å)	9.5632 (1), 17.8067 (3), 16.1958 (3)
β (°)	103.463 (1)
*V* (Å^3^)	2682.18 (7)
*Z*	4
Radiation type	Mo *K*α
μ (mm^−1^)	0.08
Crystal size (mm)	0.26 × 0.24 × 0.13

Data collection	
Diffractometer	Bruker D8 QUEST
Absorption correction	Multi-scan (*SADABS*; Krause *et al.*, 2015 ▸)
*T*_min_, *T*_max_	0.565, 0.746
No. of measured, independent and observed [*I* > 2σ(*I*)] reflections	24289, 5392, 4055
*R*_int_	0.055
(sin θ/λ)_max_ (Å^−1^)	0.622

Refinement	
*R*[*F* ^2^ > 2σ(*F* ^2^)], *wR*(*F* ^2^), *S*	0.053, 0.144, 1.06
No. of reflections	5392
No. of parameters	411
No. of restraints	155
H-atom treatment	H-atom parameters constrained
Δρ_max_, Δρ_min_ (e Å^−3^)	0.26, −0.22

Computer programs: *APEX2* and *SAINT* (Bruker, 2008 ▸), *SHELXS97* and *SHELXL97* (Sheldrick, 2008 ▸), *SHELXL2018/3* (Sheldrick, 2015 ▸), *ORTEP-3 for Windows* (Farrugia, 2012 ▸), *Mercury* (Macrae *et al.*, 2020 ▸) and *PLATON* (Spek, 2020 ▸).

## supplementary crystallographic information

### Crystal data

**Table:**

C_34_H_30_N_2_O_3_	*F*(000) = 1088
*M**_r_* = 514.60	*D*_x_ = 1.274 Mg m^−^^3^
Monoclinic, *P*2_1_/*c*	Mo *K*α radiation, λ = 0.71073 Å
*a* = 9.5632 (1) Å	Cell parameters from 4055 reflections
*b* = 17.8067 (3) Å	θ = 2.3–26.3°
*c* = 16.1958 (3) Å	µ = 0.08 mm^−^^1^
β = 103.463 (1)°	*T* = 294 K
*V* = 2682.18 (7) Å^3^	Block, colourless
*Z* = 4	0.26 × 0.24 × 0.13 mm

### Data collection

**Table:**

Bruker D8 QUEST diffractometer	4055 reflections with *I* > 2σ(*I*)
Radiation source: PHOTON-100	*R*_int_ = 0.055
ω and φ scans	θ_max_ = 26.3°, θ_min_ = 2.3°
Absorption correction: multi-scan (*SADABS*; Krause *et al.*, 2015)	*h* = −11→11
*T*_min_ = 0.565, *T*_max_ = 0.746	*k* = −22→22
24289 measured reflections	*l* = −20→19
5392 independent reflections	

### Refinement

**Table:**

Refinement on *F*^2^	Hydrogen site location: inferred from neighbouring sites
Least-squares matrix: full	H-atom parameters constrained
*R*[*F*^2^ > 2σ(*F*^2^)] = 0.053	*w* = 1/[σ^2^(*F*_o_^2^) + (0.0587*P*)^2^ + 0.8201*P*] where *P* = (*F*_o_^2^ + 2*F*_c_^2^)/3
*wR*(*F*^2^) = 0.144	(Δ/σ)_max_ = 0.001
*S* = 1.06	Δρ_max_ = 0.26 e Å^−^^3^
5392 reflections	Δρ_min_ = −0.22 e Å^−^^3^
411 parameters	Extinction correction: SHELXL2018/3 (Sheldrick 2015), Fc^*^=kFc[1+0.001xFc^2^λ^3^/sin(2θ)]^-1/4^
155 restraints	Extinction coefficient: 0.0198 (18)

### Special details

**Table:**

Geometry. All esds (except the esd in the dihedral angle between two l.s. planes) are estimated using the full covariance matrix. The cell esds are taken into account individually in the estimation of esds in distances, angles and torsion angles; correlations between esds in cell parameters are only used when they are defined by crystal symmetry. An approximate (isotropic) treatment of cell esds is used for estimating esds involving l.s. planes.

### Fractional atomic coordinates and isotropic or equivalent isotropic displacement parameters (Å^2^)

**Table:**

	*x*	*y*	*z*	*U*_iso_*/*U*_eq_	Occ. (<1)
C1	0.45235 (17)	0.17258 (8)	0.65452 (10)	0.0364 (4)	
C2	0.5169 (2)	0.13834 (10)	0.58323 (12)	0.0478 (4)	
C3	0.2724 (2)	0.12535 (11)	0.54118 (12)	0.0564 (5)	
C4	0.1366 (3)	0.10549 (16)	0.49517 (17)	0.0892 (8)	
H4	0.123305	0.082582	0.442303	0.107*	
C5	0.0218 (3)	0.12087 (19)	0.5305 (2)	0.0981 (9)	
H5	−0.070659	0.108839	0.500441	0.118*	
C6	0.0409 (2)	0.15319 (15)	0.60827 (19)	0.0800 (7)	
H6	−0.038455	0.162177	0.630907	0.096*	
C7	0.1778 (2)	0.17310 (11)	0.65468 (14)	0.0553 (5)	
H7	0.190555	0.195187	0.707949	0.066*	
C8	0.29367 (18)	0.15934 (9)	0.61985 (11)	0.0423 (4)	
C9	0.50774 (16)	0.25579 (8)	0.67048 (10)	0.0348 (3)	
H9	0.571274	0.266515	0.632400	0.042*	
C10	0.59873 (16)	0.25791 (8)	0.76320 (10)	0.0346 (3)	
H10	0.534885	0.273687	0.799327	0.042*	
C11	0.63486 (17)	0.17511 (8)	0.78236 (10)	0.0372 (4)	
H11	0.713397	0.160425	0.756183	0.045*	
C12	0.67101 (18)	0.15111 (9)	0.87455 (11)	0.0414 (4)	
C13	0.74885 (19)	0.19684 (11)	0.93799 (11)	0.0511 (4)	
H13	0.778245	0.244022	0.924110	0.061*	
C14	0.7837 (2)	0.17333 (13)	1.02196 (13)	0.0617 (5)	
H14	0.834330	0.204991	1.064128	0.074*	
C15	0.7430 (2)	0.10303 (13)	1.04259 (13)	0.0659 (6)	
H15	0.767707	0.086545	1.098588	0.079*	
C16	0.6659 (2)	0.05745 (12)	0.98034 (13)	0.0637 (6)	
H16	0.638727	0.010029	0.994909	0.076*	
C17	0.6272 (2)	0.08009 (10)	0.89584 (12)	0.0507 (4)	
C18	0.5325 (3)	0.03000 (11)	0.83073 (13)	0.0681 (6)	
H18A	0.438282	0.027019	0.843204	0.082*	
H18B	0.573028	−0.020202	0.835908	0.082*	
C19	0.5152 (2)	0.05639 (9)	0.74011 (12)	0.0531 (5)	
H19A	0.597674	0.041031	0.718941	0.064*	
H19B	0.429728	0.034133	0.704261	0.064*	
C20	0.38421 (17)	0.31188 (9)	0.65193 (11)	0.0405 (4)	
C21	0.31331 (18)	0.32852 (10)	0.56177 (11)	0.0442 (4)	
C22	0.3755 (2)	0.31242 (12)	0.49476 (12)	0.0568 (5)	
H22	0.466022	0.290241	0.505273	0.068*	
C23	0.3042 (2)	0.32906 (17)	0.41218 (14)	0.0801 (7)	
H23	0.346605	0.317979	0.367452	0.096*	
C24	0.1710 (3)	0.36189 (19)	0.39643 (16)	0.0921 (9)	
H24	0.123408	0.373183	0.340935	0.111*	
C25	0.1074 (2)	0.37819 (18)	0.46183 (17)	0.0871 (8)	
H25	0.016691	0.400127	0.450662	0.105*	
C26	0.1781 (2)	0.36202 (13)	0.54426 (15)	0.0650 (6)	
H26	0.134985	0.373605	0.588563	0.078*	
C27	0.7190 (15)	0.3150 (7)	0.7744 (19)	0.0357 (9)	0.638 (6)
C28	0.7031 (10)	0.3858 (5)	0.8046 (9)	0.0496 (12)	0.638 (6)
H28	0.619848	0.397017	0.822432	0.060*	0.638 (6)
C29	0.8066 (7)	0.4410 (4)	0.8093 (6)	0.0575 (14)	0.638 (6)
H29	0.793034	0.488124	0.830907	0.069*	0.638 (6)
C30	0.9290 (6)	0.4261 (3)	0.7822 (4)	0.0445 (13)	0.638 (6)
C31	0.9504 (6)	0.3562 (3)	0.7532 (5)	0.0490 (13)	0.638 (6)
H31	1.035161	0.345029	0.736969	0.059*	0.638 (6)
C32	0.8453 (7)	0.3019 (3)	0.7481 (7)	0.0489 (13)	0.638 (6)
H32	0.859666	0.254900	0.726375	0.059*	0.638 (6)
O3	1.0252 (4)	0.4834 (2)	0.7876 (2)	0.0793 (11)	0.638 (6)
C33	1.1100 (5)	0.4875 (3)	0.7290 (3)	0.0908 (16)	0.638 (6)
H33A	1.052694	0.475989	0.673456	0.136*	0.638 (6)
H33B	1.187392	0.452012	0.743852	0.136*	0.638 (6)
H33C	1.148553	0.537251	0.729088	0.136*	0.638 (6)
C27D	0.731 (3)	0.3074 (12)	0.770 (3)	0.0357 (9)	0.362 (6)
C28D	0.7249 (19)	0.3815 (10)	0.7913 (17)	0.0496 (12)	0.362 (6)
H28D	0.641424	0.400216	0.803831	0.060*	0.362 (6)
C29D	0.8417 (12)	0.4297 (6)	0.7949 (9)	0.049 (2)	0.362 (6)
H29D	0.835755	0.479685	0.810273	0.059*	0.362 (6)
C30D	0.9633 (9)	0.4034 (5)	0.7761 (8)	0.041 (2)	0.362 (6)
C31D	0.9735 (11)	0.3295 (5)	0.7567 (10)	0.050 (2)	0.362 (6)
H31D	1.057299	0.311310	0.744188	0.061*	0.362 (6)
C32D	0.8595 (15)	0.2815 (6)	0.7556 (13)	0.0489 (13)	0.362 (6)
H32D	0.869624	0.230713	0.745157	0.059*	0.362 (6)
O3D	1.0812 (5)	0.4476 (3)	0.7781 (4)	0.0670 (17)	0.362 (6)
C33D	1.0613 (13)	0.5235 (6)	0.7809 (10)	0.132 (4)	0.362 (6)
H33D	1.151938	0.548557	0.786794	0.198*	0.362 (6)
H33E	1.021434	0.535591	0.828434	0.198*	0.362 (6)
H33F	0.996448	0.539583	0.729374	0.198*	0.362 (6)
C34	0.4232 (4)	0.08287 (19)	0.43976 (16)	0.1045 (10)	
H34A	0.377044	0.115249	0.394113	0.157*	
H34B	0.380274	0.033877	0.431461	0.157*	
H34C	0.523694	0.079156	0.440781	0.157*	
N1	0.4056 (2)	0.11377 (10)	0.52023 (10)	0.0611 (5)	
N2	0.50281 (15)	0.13804 (7)	0.73799 (8)	0.0379 (3)	
O1	0.64375 (16)	0.13463 (9)	0.58338 (10)	0.0689 (4)	
O2	0.34147 (16)	0.34041 (8)	0.70952 (9)	0.0624 (4)	

### Atomic displacement parameters (Å^2^)

**Table:**

	*U*^11^	*U*^22^	*U*^33^	*U*^12^	*U*^13^	*U*^23^
C1	0.0427 (8)	0.0343 (8)	0.0314 (8)	0.0025 (6)	0.0071 (7)	−0.0015 (6)
C2	0.0607 (11)	0.0420 (9)	0.0424 (10)	0.0081 (8)	0.0155 (9)	−0.0025 (8)
C3	0.0646 (12)	0.0542 (11)	0.0428 (11)	−0.0016 (9)	−0.0030 (9)	−0.0054 (9)
C4	0.0880 (19)	0.100 (2)	0.0612 (15)	−0.0108 (15)	−0.0211 (13)	−0.0222 (14)
C5	0.0588 (15)	0.115 (2)	0.104 (2)	−0.0155 (14)	−0.0164 (15)	−0.0115 (18)
C6	0.0485 (12)	0.0855 (17)	0.102 (2)	−0.0072 (11)	0.0103 (12)	0.0003 (15)
C7	0.0472 (10)	0.0543 (11)	0.0634 (13)	−0.0030 (8)	0.0108 (9)	0.0003 (9)
C8	0.0474 (9)	0.0361 (8)	0.0397 (9)	−0.0032 (7)	0.0027 (7)	0.0009 (7)
C9	0.0364 (8)	0.0346 (8)	0.0334 (8)	0.0012 (6)	0.0079 (6)	0.0016 (6)
C10	0.0392 (8)	0.0330 (8)	0.0314 (8)	0.0012 (6)	0.0076 (6)	0.0003 (6)
C11	0.0404 (8)	0.0337 (8)	0.0362 (9)	0.0031 (6)	0.0062 (7)	−0.0011 (7)
C12	0.0428 (9)	0.0410 (9)	0.0381 (9)	0.0065 (7)	0.0049 (7)	0.0045 (7)
C13	0.0500 (10)	0.0578 (11)	0.0413 (10)	−0.0048 (8)	0.0019 (8)	0.0049 (8)
C14	0.0592 (12)	0.0800 (15)	0.0398 (11)	−0.0069 (10)	−0.0010 (9)	0.0024 (10)
C15	0.0699 (13)	0.0818 (15)	0.0403 (11)	0.0028 (11)	0.0013 (9)	0.0189 (10)
C16	0.0803 (14)	0.0555 (12)	0.0516 (12)	0.0036 (10)	0.0081 (10)	0.0180 (10)
C17	0.0626 (11)	0.0406 (9)	0.0453 (10)	0.0065 (8)	0.0052 (8)	0.0074 (8)
C18	0.1057 (17)	0.0350 (9)	0.0558 (13)	−0.0070 (10)	0.0028 (12)	0.0072 (9)
C19	0.0723 (12)	0.0310 (9)	0.0506 (11)	0.0010 (8)	0.0031 (9)	−0.0033 (8)
C20	0.0429 (9)	0.0334 (8)	0.0449 (10)	0.0006 (7)	0.0098 (7)	0.0026 (7)
C21	0.0399 (9)	0.0421 (9)	0.0484 (10)	−0.0010 (7)	0.0055 (7)	0.0099 (8)
C22	0.0462 (10)	0.0761 (13)	0.0460 (11)	0.0065 (9)	0.0064 (8)	0.0113 (10)
C23	0.0601 (13)	0.133 (2)	0.0449 (12)	0.0098 (13)	0.0070 (10)	0.0145 (13)
C24	0.0613 (14)	0.155 (3)	0.0520 (14)	0.0119 (15)	−0.0034 (11)	0.0281 (15)
C25	0.0482 (12)	0.130 (2)	0.0766 (17)	0.0222 (13)	0.0007 (11)	0.0321 (16)
C26	0.0499 (11)	0.0825 (15)	0.0617 (13)	0.0149 (10)	0.0107 (10)	0.0182 (11)
C27	0.041 (2)	0.037 (2)	0.027 (3)	0.000 (2)	0.0048 (18)	0.000 (2)
C28	0.041 (3)	0.0396 (13)	0.071 (5)	0.0006 (17)	0.0188 (16)	−0.0077 (15)
C29	0.043 (3)	0.042 (2)	0.090 (4)	−0.0038 (19)	0.019 (2)	−0.023 (2)
C30	0.038 (3)	0.045 (3)	0.049 (2)	−0.007 (2)	0.006 (2)	−0.009 (2)
C31	0.046 (2)	0.050 (3)	0.054 (2)	−0.002 (2)	0.019 (2)	−0.007 (3)
C32	0.0555 (18)	0.039 (3)	0.057 (2)	−0.002 (2)	0.0224 (17)	−0.007 (3)
O3	0.068 (2)	0.062 (2)	0.111 (2)	−0.0330 (17)	0.0269 (16)	−0.0194 (18)
C33	0.073 (3)	0.074 (3)	0.136 (4)	−0.025 (2)	0.046 (3)	0.007 (3)
C27D	0.041 (2)	0.037 (2)	0.027 (3)	0.000 (2)	0.0048 (18)	0.000 (2)
C28D	0.041 (3)	0.0396 (13)	0.071 (5)	0.0006 (17)	0.0188 (16)	−0.0077 (15)
C29D	0.047 (5)	0.029 (3)	0.076 (5)	0.002 (3)	0.023 (4)	−0.007 (3)
C30D	0.031 (3)	0.045 (5)	0.045 (4)	0.002 (3)	0.006 (3)	−0.001 (4)
C31D	0.041 (3)	0.048 (5)	0.067 (4)	0.005 (3)	0.020 (3)	−0.006 (5)
C32D	0.0555 (18)	0.039 (3)	0.057 (2)	−0.002 (2)	0.0224 (17)	−0.007 (3)
O3D	0.040 (2)	0.052 (3)	0.106 (4)	−0.0002 (19)	0.010 (2)	0.007 (3)
C33D	0.104 (6)	0.075 (5)	0.215 (9)	−0.006 (5)	0.034 (6)	0.009 (6)
C34	0.148 (3)	0.121 (2)	0.0454 (14)	0.018 (2)	0.0227 (15)	−0.0299 (15)
N1	0.0810 (12)	0.0650 (11)	0.0350 (9)	0.0052 (9)	0.0087 (8)	−0.0150 (7)
N2	0.0476 (7)	0.0300 (7)	0.0331 (7)	−0.0004 (5)	0.0032 (6)	−0.0005 (5)
O1	0.0658 (9)	0.0790 (10)	0.0696 (10)	0.0142 (7)	0.0309 (8)	−0.0106 (8)
O2	0.0726 (9)	0.0613 (9)	0.0524 (8)	0.0279 (7)	0.0125 (7)	−0.0047 (7)

### Geometric parameters (Å, º)

**Table:**

C1—N2	1.461 (2)	C21—C22	1.384 (3)
C1—C8	1.509 (2)	C21—C26	1.392 (3)
C1—C2	1.555 (2)	C22—C23	1.385 (3)
C1—C9	1.575 (2)	C22—H22	0.9300
C2—O1	1.214 (2)	C23—C24	1.371 (3)
C2—N1	1.364 (3)	C23—H23	0.9300
C3—C8	1.382 (3)	C24—C25	1.369 (4)
C3—C4	1.384 (3)	C24—H24	0.9300
C3—N1	1.408 (3)	C25—C26	1.379 (3)
C4—C5	1.378 (4)	C25—H25	0.9300
C4—H4	0.9300	C26—H26	0.9300
C5—C6	1.358 (4)	C27—C28	1.373 (7)
C5—H5	0.9300	C27—C32	1.390 (8)
C6—C7	1.396 (3)	C28—C29	1.385 (7)
C6—H6	0.9300	C28—H28	0.9300
C7—C8	1.377 (3)	C29—C30	1.369 (5)
C7—H7	0.9300	C29—H29	0.9300
C9—C20	1.523 (2)	C30—C31	1.362 (6)
C9—C10	1.551 (2)	C30—O3	1.363 (5)
C9—H9	0.9800	C31—C32	1.383 (6)
C10—C27	1.514 (10)	C31—H31	0.9300
C10—C27D	1.522 (17)	C32—H32	0.9300
C10—C11	1.529 (2)	O3—C33	1.386 (6)
C10—H10	0.9800	C33—H33A	0.9600
C11—N2	1.457 (2)	C33—H33B	0.9600
C11—C12	1.514 (2)	C33—H33C	0.9600
C11—H11	0.9800	C27D—C28D	1.369 (12)
C12—C13	1.385 (3)	C27D—C32D	1.383 (12)
C12—C17	1.400 (2)	C28D—C29D	1.399 (12)
C13—C14	1.387 (3)	C28D—H28D	0.9300
C13—H13	0.9300	C29D—C30D	1.353 (9)
C14—C15	1.375 (3)	C29D—H29D	0.9300
C14—H14	0.9300	C30D—C31D	1.361 (9)
C15—C16	1.368 (3)	C30D—O3D	1.370 (9)
C15—H15	0.9300	C31D—C32D	1.382 (12)
C16—C17	1.392 (3)	C31D—H31D	0.9300
C16—H16	0.9300	C32D—H32D	0.9300
C17—C18	1.511 (3)	O3D—C33D	1.367 (11)
C18—C19	1.513 (3)	C33D—H33D	0.9600
C18—H18A	0.9700	C33D—H33E	0.9600
C18—H18B	0.9700	C33D—H33F	0.9600
C19—N2	1.459 (2)	C34—N1	1.460 (3)
C19—H19A	0.9700	C34—H34A	0.9600
C19—H19B	0.9700	C34—H34B	0.9600
C20—O2	1.214 (2)	C34—H34C	0.9600
C20—C21	1.490 (2)		
			
N2—C1—C8	111.44 (13)	C22—C21—C26	118.54 (17)
N2—C1—C2	114.79 (13)	C22—C21—C20	123.15 (15)
C8—C1—C2	101.60 (13)	C26—C21—C20	118.31 (17)
N2—C1—C9	102.60 (12)	C21—C22—C23	120.53 (19)
C8—C1—C9	118.61 (13)	C21—C22—H22	119.7
C2—C1—C9	108.32 (13)	C23—C22—H22	119.7
O1—C2—N1	126.10 (17)	C24—C23—C22	119.9 (2)
O1—C2—C1	126.08 (17)	C24—C23—H23	120.0
N1—C2—C1	107.82 (15)	C22—C23—H23	120.0
C8—C3—C4	121.8 (2)	C25—C24—C23	120.5 (2)
C8—C3—N1	109.83 (16)	C25—C24—H24	119.8
C4—C3—N1	128.4 (2)	C23—C24—H24	119.8
C5—C4—C3	117.6 (2)	C24—C25—C26	119.9 (2)
C5—C4—H4	121.2	C24—C25—H25	120.0
C3—C4—H4	121.2	C26—C25—H25	120.0
C6—C5—C4	121.4 (2)	C25—C26—C21	120.6 (2)
C6—C5—H5	119.3	C25—C26—H26	119.7
C4—C5—H5	119.3	C21—C26—H26	119.7
C5—C6—C7	120.9 (2)	C28—C27—C32	116.1 (7)
C5—C6—H6	119.5	C28—C27—C10	121.0 (6)
C7—C6—H6	119.5	C32—C27—C10	122.7 (7)
C8—C7—C6	118.5 (2)	C27—C28—C29	122.3 (5)
C8—C7—H7	120.7	C27—C28—H28	118.8
C6—C7—H7	120.7	C29—C28—H28	118.8
C7—C8—C3	119.72 (17)	C30—C29—C28	119.9 (5)
C7—C8—C1	130.92 (16)	C30—C29—H29	120.1
C3—C8—C1	109.32 (16)	C28—C29—H29	120.1
C20—C9—C10	114.19 (13)	C31—C30—O3	123.6 (5)
C20—C9—C1	111.66 (12)	C31—C30—C29	119.7 (4)
C10—C9—C1	105.86 (12)	O3—C30—C29	116.7 (5)
C20—C9—H9	108.3	C30—C31—C32	119.6 (5)
C10—C9—H9	108.3	C30—C31—H31	120.2
C1—C9—H9	108.3	C32—C31—H31	120.2
C27—C10—C11	119.6 (7)	C31—C32—C27	122.4 (6)
C27D—C10—C11	113.6 (12)	C31—C32—H32	118.8
C27—C10—C9	111.8 (12)	C27—C32—H32	118.8
C27D—C10—C9	110 (2)	C30—O3—C33	119.3 (4)
C11—C10—C9	102.84 (12)	O3—C33—H33A	109.5
C27—C10—H10	107.3	O3—C33—H33B	109.5
C11—C10—H10	107.3	H33A—C33—H33B	109.5
C9—C10—H10	107.3	O3—C33—H33C	109.5
N2—C11—C12	109.33 (13)	H33A—C33—H33C	109.5
N2—C11—C10	101.99 (12)	H33B—C33—H33C	109.5
C12—C11—C10	117.46 (13)	C28D—C27D—C32D	117.0 (13)
N2—C11—H11	109.2	C28D—C27D—C10	119.6 (12)
C12—C11—H11	109.2	C32D—C27D—C10	123.5 (12)
C10—C11—H11	109.2	C27D—C28D—C29D	121.5 (11)
C13—C12—C17	119.24 (16)	C27D—C28D—H28D	119.3
C13—C12—C11	121.72 (15)	C29D—C28D—H28D	119.3
C17—C12—C11	119.02 (15)	C30D—C29D—C28D	119.9 (9)
C12—C13—C14	120.99 (18)	C30D—C29D—H29D	120.0
C12—C13—H13	119.5	C28D—C29D—H29D	120.0
C14—C13—H13	119.5	C29D—C30D—C31D	119.8 (8)
C15—C14—C13	119.7 (2)	C29D—C30D—O3D	122.8 (9)
C15—C14—H14	120.1	C31D—C30D—O3D	117.3 (8)
C13—C14—H14	120.1	C30D—C31D—C32D	120.1 (9)
C16—C15—C14	119.70 (19)	C30D—C31D—H31D	119.9
C16—C15—H15	120.2	C32D—C31D—H31D	119.9
C14—C15—H15	120.2	C31D—C32D—C27D	121.5 (11)
C15—C16—C17	121.8 (2)	C31D—C32D—H32D	119.3
C15—C16—H16	119.1	C27D—C32D—H32D	119.3
C17—C16—H16	119.1	C33D—O3D—C30D	116.6 (8)
C16—C17—C12	118.50 (18)	O3D—C33D—H33D	109.5
C16—C17—C18	119.64 (18)	O3D—C33D—H33E	109.5
C12—C17—C18	121.76 (16)	H33D—C33D—H33E	109.5
C17—C18—C19	113.98 (17)	O3D—C33D—H33F	109.5
C17—C18—H18A	108.8	H33D—C33D—H33F	109.5
C19—C18—H18A	108.8	H33E—C33D—H33F	109.5
C17—C18—H18B	108.8	N1—C34—H34A	109.5
C19—C18—H18B	108.8	N1—C34—H34B	109.5
H18A—C18—H18B	107.7	H34A—C34—H34B	109.5
N2—C19—C18	108.81 (15)	N1—C34—H34C	109.5
N2—C19—H19A	109.9	H34A—C34—H34C	109.5
C18—C19—H19A	109.9	H34B—C34—H34C	109.5
N2—C19—H19B	109.9	C2—N1—C3	111.40 (15)
C18—C19—H19B	109.9	C2—N1—C34	123.9 (2)
H19A—C19—H19B	108.3	C3—N1—C34	124.7 (2)
O2—C20—C21	120.82 (15)	C11—N2—C19	112.57 (13)
O2—C20—C9	120.50 (15)	C11—N2—C1	109.09 (12)
C21—C20—C9	118.63 (14)	C19—N2—C1	116.67 (13)
			
N2—C1—C2—O1	−58.2 (2)	C9—C20—C21—C22	−18.0 (2)
C8—C1—C2—O1	−178.62 (18)	O2—C20—C21—C26	−15.4 (3)
C9—C1—C2—O1	55.7 (2)	C9—C20—C21—C26	162.11 (17)
N2—C1—C2—N1	122.25 (16)	C26—C21—C22—C23	−0.3 (3)
C8—C1—C2—N1	1.87 (18)	C20—C21—C22—C23	179.9 (2)
C9—C1—C2—N1	−123.79 (15)	C21—C22—C23—C24	0.1 (4)
C8—C3—C4—C5	0.1 (4)	C22—C23—C24—C25	−0.2 (5)
N1—C3—C4—C5	178.0 (2)	C23—C24—C25—C26	0.5 (5)
C3—C4—C5—C6	−1.1 (5)	C24—C25—C26—C21	−0.6 (4)
C4—C5—C6—C7	1.0 (5)	C22—C21—C26—C25	0.5 (3)
C5—C6—C7—C8	0.1 (4)	C20—C21—C26—C25	−179.6 (2)
C6—C7—C8—C3	−1.0 (3)	C11—C10—C27—C28	−143.2 (18)
C6—C7—C8—C1	−178.42 (19)	C9—C10—C27—C28	97 (2)
C4—C3—C8—C7	1.0 (3)	C11—C10—C27—C32	43 (3)
N1—C3—C8—C7	−177.27 (17)	C9—C10—C27—C32	−77 (2)
C4—C3—C8—C1	178.9 (2)	C32—C27—C28—C29	0 (3)
N1—C3—C8—C1	0.6 (2)	C10—C27—C28—C29	−174.9 (16)
N2—C1—C8—C7	53.4 (2)	C27—C28—C29—C30	1 (2)
C2—C1—C8—C7	176.10 (18)	C28—C29—C30—C31	−2.2 (13)
C9—C1—C8—C7	−65.4 (2)	C28—C29—C30—O3	179.1 (9)
N2—C1—C8—C3	−124.20 (15)	O3—C30—C31—C32	−178.6 (7)
C2—C1—C8—C3	−1.48 (18)	C29—C30—C31—C32	2.8 (11)
C9—C1—C8—C3	117.03 (16)	C30—C31—C32—C27	−2.3 (19)
N2—C1—C9—C20	−119.90 (14)	C28—C27—C32—C31	1 (3)
C8—C1—C9—C20	3.37 (19)	C10—C27—C32—C31	175.4 (15)
C2—C1—C9—C20	118.32 (15)	C31—C30—O3—C33	32.0 (9)
N2—C1—C9—C10	4.93 (15)	C29—C30—O3—C33	−149.3 (7)
C8—C1—C9—C10	128.20 (14)	C11—C10—C27D—C28D	−151 (3)
C2—C1—C9—C10	−116.85 (14)	C9—C10—C27D—C28D	94 (4)
C20—C9—C10—C27	−87.6 (4)	C11—C10—C27D—C32D	28 (5)
C1—C9—C10—C27	149.2 (4)	C9—C10—C27D—C32D	−87 (4)
C20—C9—C10—C27D	−95.6 (7)	C32D—C27D—C28D—C29D	3 (6)
C1—C9—C10—C27D	141.2 (7)	C10—C27D—C28D—C29D	−178 (3)
C20—C9—C10—C11	142.92 (13)	C27D—C28D—C29D—C30D	1 (4)
C1—C9—C10—C11	19.68 (15)	C28D—C29D—C30D—C31D	−2 (2)
C27—C10—C11—N2	−161.6 (11)	C28D—C29D—C30D—O3D	179.7 (16)
C27D—C10—C11—N2	−156 (2)	C29D—C30D—C31D—C32D	0 (2)
C9—C10—C11—N2	−37.08 (14)	O3D—C30D—C31D—C32D	178.5 (14)
C27—C10—C11—C12	78.9 (11)	C30D—C31D—C32D—C27D	3 (4)
C27D—C10—C11—C12	84 (2)	C28D—C27D—C32D—C31D	−5 (6)
C9—C10—C11—C12	−156.53 (13)	C10—C27D—C32D—C31D	176 (3)
N2—C11—C12—C13	−152.60 (16)	C29D—C30D—O3D—C33D	−14.6 (17)
C10—C11—C12—C13	−37.1 (2)	C31D—C30D—O3D—C33D	167.5 (12)
N2—C11—C12—C17	28.8 (2)	O1—C2—N1—C3	178.84 (19)
C10—C11—C12—C17	144.31 (16)	C1—C2—N1—C3	−1.6 (2)
C17—C12—C13—C14	−0.1 (3)	O1—C2—N1—C34	−3.9 (3)
C11—C12—C13—C14	−178.63 (17)	C1—C2—N1—C34	175.6 (2)
C12—C13—C14—C15	1.3 (3)	C8—C3—N1—C2	0.7 (2)
C13—C14—C15—C16	−1.3 (3)	C4—C3—N1—C2	−177.4 (2)
C14—C15—C16—C17	0.1 (4)	C8—C3—N1—C34	−176.5 (2)
C15—C16—C17—C12	1.1 (3)	C4—C3—N1—C34	5.4 (4)
C15—C16—C17—C18	−175.4 (2)	C12—C11—N2—C19	−60.40 (17)
C13—C12—C17—C16	−1.1 (3)	C10—C11—N2—C19	174.56 (13)
C11—C12—C17—C16	177.49 (17)	C12—C11—N2—C1	168.44 (12)
C13—C12—C17—C18	175.28 (19)	C10—C11—N2—C1	43.40 (15)
C11—C12—C17—C18	−6.1 (3)	C18—C19—N2—C11	66.5 (2)
C16—C17—C18—C19	−172.27 (19)	C18—C19—N2—C1	−166.23 (16)
C12—C17—C18—C19	11.4 (3)	C8—C1—N2—C11	−158.00 (13)
C17—C18—C19—N2	−39.1 (3)	C2—C1—N2—C11	87.21 (16)
C10—C9—C20—O2	−17.6 (2)	C9—C1—N2—C11	−30.05 (15)
C1—C9—C20—O2	102.41 (18)	C8—C1—N2—C19	73.08 (18)
C10—C9—C20—C21	164.84 (13)	C2—C1—N2—C19	−41.7 (2)
C1—C9—C20—C21	−75.12 (17)	C9—C1—N2—C19	−158.97 (14)
O2—C20—C21—C22	164.45 (18)		

### Hydrogen-bond geometry (Å, º)

**Table:**

*D*—H···*A*	*D*—H	H···*A*	*D*···*A*	*D*—H···*A*
C33—H33*B*···O2^i^	0.96	2.61	3.490 (5)	153
C4—H4···O3*D*^ii^	0.93	2.65	3.559 (6)	166

Symmetry codes: (i) *x*+1, *y*, *z*; (ii) *x*−1, −*y*+1/2, *z*−1/2.
